# Correction: Differential pathogenesis of Usutu virus isolates in mice

**DOI:** 10.1371/journal.pntd.0010515

**Published:** 2022-06-02

**Authors:** Sarah C. Kuchinsky, Seth A. Hawks, Eric C. Mossel, Sheryl Coutermarsh-Ott, Nisha K. Duggal

The virus isolate UG09615 is incorrectly reported throughout the article as isolated in 2010 in Uganda. The correct date of isolation in Uganda is 2012. The date of isolate collection has been updated in the reference GenBank sequence with accession #MN813491.
