# Supplementary material for: Differential pathogenesis of Usutu virus isolates in mice
Source: PLoS Negl Trop Dis. 2020 Oct 12;14(10):e0008765. doi: 10.1371/journal.pntd.0008765 (PMC7580916; doi:10.1371/journal.pntd.0008765)
Supplement: S1 Table — Amino acid differences are highlighted in bold text. (DOCX) [file pntd.0008765.s001.docx]

**Supplementary Table 1.**

| **Amino acid substitutions compared to South Africa 1959** | | | | | | |
| --- | --- | --- | --- | --- | --- | --- |
| Protein | Amino acid | South Africa 1959 | Spain 2009 | Netherlands 2016 | Uganda 2010 | Senegal 2003 |
| C | 101 | K | **R** | K | K | K |
|  | 105 | G | G | **S** | G | G |
|  | 112 | V | **L** | **L** | V | V |
|  | 123 | M | **I** | M | M | M |
| prM | 153 | T | **M** | T | T | T |
|  | 172 | D | **G** | D | D | D |
|  | 215 | R | **L** | R | R | R |
|  | 283 | V | V | V | V | **I** |
| E | 345 | N | N | **S** | N | N |
|  | 381 | D | D | **N** | D | D |
|  | 386 | K | **R** | K | K | K |
|  | 472 | K | K | **E** | K | K |
|  | 506 | V | V | V | **I** | V |
|  | 524 | S | S | **L** | S | S |
|  | 531 | I | I | **T** | I | I |
|  | 569 | S | **G** | **G** | **G** | **G** |
|  | 585 | E | E | **K** | E | E |
|  | 595 | G | **S** | G | G | G |
|  | 613 | S | **G** | **G** | **G** | **G** |
|  | 637 | S | S | **T** | S | S |
|  | 790 | S | **N** | **N** | **N** | **N** |
| NS1 | 843 | H | H | **Y** | H | H |
|  | 891 | K | K | K | **R** | **R** |
|  | 900 | T | T | T | T | **I** |
|  | 939 | V | V | V | V | **A** |
|  | 967 | H | **Y** | H | H | H |
|  | 1067 | V | **I** | V | V | V |
|  | 1117 | K | **R** | **R** | **R** | **R** |
| NS2A | 1190 | I | I | I | I | **V** |
|  | 1227 | A | **V** | A | A | A |
|  | 1236 | A | **T** | A | A | A |
|  | 1240 | L | L | L | L | **F** |
|  | 1267 | D | **N** | **N** | **N** | **N** |
|  | 1268 | L | **F** | L | **F** | **F** |
|  | 1270 | L | **F** | L | L | L |
|  | 1287 | A | A | **V** | A | A |
|  | 1322 | I | I | **V** | I | I |
|  | 1334 | A | A | **V** | A | A |
| NS2B | 1436 | T | **A** | T | T | **A** |
|  | 1460 | I | **V** | I | I | I |
|  | 1492 | I | I | I | I | **V** |
| NS3 | 1549 | L | L | **F** | L | L |
|  | 1602 | I | I | **V** | I | I |
|  | 1618 | V | V | **I** | **I** | **I** |
|  | 1645 | K | **R** | K | K | K |
|  | 1981 | G | **S** | G | G | G |
|  | 1983 | S | S | S | **N** | S |
|  | 2059 | I | I | **V** | I | I |
|  | 2075 | I | **V** | I | I | I |
|  | 2106 | S | S | **A** | **A** | S |
| NS4B | 2290 | G | **S** | **S** | **S** | **S** |
|  | 2294 | P | **S** | P | P | P |
|  | 2301 | P | **H** | P | P | P |
|  | 2355 | N | **T** | N | N | N |
|  | 2393 | T | **I** | T | T | T |
|  | 2460 | L | L | **F** | L | L |
| NS5 | 2550 | R | R | **K** | **K** | R |
|  | 2552 | D | **E** | D | D | D |
|  | 2620 | A | **V** | A | A | A |
|  | 2695 | E | **D** | E | E | E |
|  | 2706 | R | R | R | R | **K** |
|  | 2765 | T | **P** | T | T | T |
|  | 2777 | T | T | T | T | **A** |
|  | 2784 | E | **D** | E | E | E |
|  | 2803 | A | A | **S** | A | A |
|  | 2849 | G | **S** | **S** | **S** | **S** |
|  | 2902 | R | **K** | R | R | R |
|  | 3060 | K | K | **R** | K | K |
|  | 3088 | M | **I** | M | M | M |
|  | 3322 | V | **I** | V | V | V |
|  | 3430 | E | E | E | E | **G** |
